# hnRNPA2B1 promotes the occurrence and progression of hepatocellular carcinoma by downregulating PCK1 mRNA via a m6A RNA methylation manner

**DOI:** 10.1186/s12967-023-04704-4

**Published:** 2023-11-28

**Authors:** Weijie Hao, Zhimin Chen, Jingzhi Tang, Ru Yang, Wei-Qiang Gao, Huiming Xu

**Affiliations:** 1 State Key Laboratory of Oncogenes and Related Genes, Renji-Med X Clinical Stem Cell Research Center, Ren Ji Hospital, School of Medicine, Shanghai Jiao Tong University, Shanghai, China; 2 Department of Ultrasound, Fudan University Shanghai Cancer Center, Shanghai, China; 3 Med-X Research Institute and School of Biological Medical Engineering, Shanghai Jiao Tong University, Shanghai, China

**Keywords:** hnRNPA2B1, Hepatocellular carcinoma, Hepatocarcinogenesis, Gluconeogenesis, PCK1

## Abstract

**Background:**

N6-methyladenosine (m6A) is the most prevalent RNA modification. Although hnRNPA2B1, as a reader of m6A modification, has been reported to promote tumorigenesis in a few types of tumors, its role in hepatocellular carcinoma (HCC) and the underlying molecular mechanism remains unclear.

**Methods:**

Multiple public databases were used to analyze the expression of hnRNPA2B1 in HCC and its correlation with survival prognosis. We employed a CRISPR-Cas9 sgRNA editing strategy to knockout hnRNPA2B1 expression in HCC cells. The biological function of hnRNPA2B1 in vitro in HCC cells was measured by CCK8, colony formation, migration, and invasion assay. The tumorigenic function of hnRNPA2B1 in vivo was determined by a subcutaneous tumor formation experiment and a HCC mouse model via tail injection of several plasmids into the mouse within 5s-7s. RNA binding protein immunoprecipitation (RIP) experiment using hnRNPA2B1 was performed to test the target genes of hnRNPA2B1 and methylated RNA immunoprecipitation (MeRIP) assay was performed to explore the m6A methylated mRNA of target genes.

**Results:**

hnRNPA2B1 highly expressed in HCC tissues, correlated with high grades and poor prognosis. Its knockout reduced HCC cell proliferation, migration, and invasion in vitro, while overexpression promoted these processes. hnRNPA2B1-knockout cells inhibited tumor formation in graft experiments. In HCC mice, endogenous knockout attenuated hepatocarcinogenesis. RNA-seq showed downregulated gluconeogenesis with high hnRNPA2B1 expression. hnRNPA2B1 negatively correlated with PCK1, a key enzyme. RIP assay revealed hnRNPA2B1 binding to PCK1 mRNA. hnRNPA2B1 knockout increased m6A-methylation of PCK1 mRNA. Interestingly, PCK1 knockout partially counteracted tumor inhibition by hnRNPA2B1 knockout in mice.

**Conclusion:**

Our study indicated that hnRNPA2B1 is highly expressed in HCC and correlated with a poor prognosis. hnRNPA2B1 promotes the tumorigenesis and progression of HCC both in vitro and in vivo. Moreover, hnRNPA2B1 downregulates the expression of PCK1 mRNA via a m6A methylation manner. More importantly, the ability of hnRNPA2B1 to induce tumorigenesis and progression in HCC is dependent on its ability to decrease the expression of PCK1. Therefore, this study suggested that hnRNPA2B1 might be a diagnostic marker of poor prognosis of HCC and a potential therapeutic target for HCC patients.

**Supplementary Information:**

The online version contains supplementary material available at 10.1186/s12967-023-04704-4.

## Introduction

Liver cancer is one of the most common cancers with increasing incidence and mortality rates [1]. Hepatocellular carcinoma (HCC) accounts for the majority of primary liver cancers [2]. Despite the advancements in the combination of surgical resection and chemotherapy for HCC patients, the overall survival rate remains below 2 years [3]. Recurrence and metastasis are the main causes of death in 90% of patients [4]. Targeted therapy or immunotherapy may offer effective treatments by targeting specific genes or pathways [5]. Therefore, further investigation of the molecular mechanisms of HCC is desired.

N^6^-methyladenosine (m6A) is the most common and abundant modification on different types of RNAs in eukaryotes [6]. The m6A modification has been demonstrated to affect various aspects of RNA metabolism including pre-mRNA splicing, 3′-end processing, maturation, nuclear export, localization, translation, RNA stability, and non-coding RNA processing [7, 8]. m6A modification plays a crucial role in the normal physiological process and pathological progression, especially in cancer. m6A modification is a dynamic and reversible process regulated by m6A methyltransferases (termed “writers”), demethylases (termed “erasers”), and m6A sites recognizing and binding proteins (termed “readers”). Emerging evidence suggests that m6A RNA methylation participates in the initiation and progression of cancer [9, 10]. Bioinformation analyses based on The Cancer Genome Atlas (TCGA) database reveal that m6A methylation modulators and related genes are different in HCC patients and are correlated with survival and prognosis [11, 12]. Recently, several studies have shown that abnormal m6A RNA methylation modulators are associated with HCC progression [13–15]. m6A methyltransferase-like 3 (METTL3) is upregulated in HCC and promotes HCC cell proliferation, migration, and clone formation via regulating m6A-mediated SOCS2 mRNA degradation dependent on the m6A reader protein YTHDF2 [16].

The RNA-binding protein hnRNPA2B1 is a member of the hnRNPs family proteins that serve as the main components of spliceosomes and bind to the pre-mRNA to participate in mRNA metabolism [17]. hnRNPA2B1 is involved in the regulation of RNA metabolism in many aspects including transcription, splicing processing, transport, stability, and translational regulation [18]. The expression of hnRNPA2B1 is upregulated in various tumors where it has been shown to promote tumorigenesis and progression [19, 20]. In addition, hnRNPA2B1 also regulates the sensitivity of chemotherapeutic drugs and endocrine resistance in breast cancer cells [19]. As a reader of m6A mRNA, hnRNPA2B1 enhances the m6A-dependent stabilization of mRNAs of several oncogenes and promotes cancer progression [19, 20]. However, whether hnRNPA2B1 can promote the tumorigenesis and progression of HCC and its molecular mechanism remains unclear.

In this study, we analyzed the expression of hnRNPA2B1 in HCC according to the TCGA database, then performed a correlation analysis of the expression of hnRNPA2B1 with overall survival rate and clinical pathological stages. Next, we determined the function of hnRNPA2B1 on the tumorigenesis and progression of HCC by in vitro and in vivo experiments and explored the underlying molecular mechanism. We found that hnRNPA2B1 promotes the occurrence and progression of HCC via the gluconeogenic pathway.

## Materials and methods

### Data collection

Gene expression data and corresponding clinical information were obtained from two public databases, The Cancer Genome Atlas (TCGA, https://portal.gdc.cancer.gov/) and GEO (https://www.ncbi.nlm.nih.gov/geo/). Furthermore, the cohort of Chinese patients with hepatitis B virus (HBV) infection (CHCC-HBV) included 159 patients with liver cancer who underwent primary radical resection at Zhongshan Hospital (Fudan University, Shanghai, China) between 2010 and 2014 [21]. The dataset details are shown in Additional file 1: Table S1, and the clinicopathologic characteristics of patient cohorts was calculated the significance using Table 1 (https://benjaminrich.github.io/table1/vignettes/table1-examples.html) R package are presented in Additional file 1: Table S2.

### Cell culture

HEK 293T cells, Huh7, MHCC-97H, and HepG2 HCC cell lines were obtained from the Chinese Academy of Sciences Cell Bank in Shanghai, China. The cells were cultured in DMEM (Gibco, USA) supplemented with 10% fetal bovine serum (FBS, Sigma, USA) and 1% Penicillin/Streptomycin (P/S, Gibco, USA) at 37°C in the presence of 5% CO2.

### Knockdown and overexpression of hnRNPA2B1

We constructed sgRNA targeting human hnRNPA2B1 and PCK1 plasmid, and non-sense scrambled control plasmid with lenti-CRISPR-V2 vector (Addgene #52,961) based on CRIPSR-Cas 9 strategy using the following primers presented in the Additional file 1: Table S2. In addition, we constructed overexpression plasmid of human hnRNPA2B1 and PCK1 with pCDH-CMV-GFP-Puro vector (Addgene #91,892). Empty vector pCDH-CMV-GFP-Puro was used as control. Lentiviral particles were produced in HEK293T cells with hnRNPA2B1/PCK1 sgRNA plasmid or hnRNPA2B1/PCK1 overexpression plasmid together with two package plasmids psPAX2 (Addgene #12,260) and pMD2.G (Addgene #12,259). HCC cell lines were infected with the viral supernatants. After 24 h of infection, 5 μg/ml puromycin was added for cell screening to establish a stable knockout or overexpression cell lines. Finally, the expression of hnRNPA2B1 and PCK1 were confirmed by RT-PCR and western blot. The sequences of the cloning primers and sgRNA are listed in Additional file 1: Table S3.

### Quantitative real-time PCR

Total RNA was extracted using RNA Isolation Kit (OMEGA, Norcross, USA) according to the manufacturer's instructions and reverse transcribed into cDNA using HiScript II Q Select RT SuperMix Kit (Vazyme, Nanjing, China). Quantitative real-time PCR (RT-PCR) was performed using the ChamQ Universal SYBR qPCR Master Mix Kit (Vazyme, Nanjing, China). The relative amount of each gene was quantified by the 2 − ΔΔCt method and normalized by the expression of β-actin. All quantitative RT-PCR experiments were performed in at least three independent experiments for culture cells. Primer sequences are listed in Additional file 1: Table S3.

### Western blotting

The cells were lysed with RIPA Lysis buffer (Thermo Fisher Science, USA) that contained a protein inhibitor cocktail (Merck, USA). The protein concentration was assessed using the BCA kit (Thermo Fisher, USA). The PVDF membranes were blocked with non-fat milk and then incubated with primary antibodies indicated as follows: hnRNPA2B1 rabbit antibody (1:1000, #A1162, Abclonal, China), PCK1 rabbit antibody (1:1000, #A22172, ABclonal, China), β-Actin Mouse antibody, (1:1000, #AC004, ABclonal, China), Glycolysis Antibody Sampler Kit (#8337, Cell Signaling Technology, USA). After being washed with TBST, the membranes were incubated with corresponding HRP-conjugated secondary antibodies. The membranes were visualized with the BioRad VersaDoc 4000 imaging system, and densitometric analysis of proteins was quantified by Quantity One software (BioRad, CA, USA).

### Immunohistochemistry staining

The liver cancer tissues from the mice were fixed, embedded in paraffin, and cut into 5 μm thick sections for immunohistochemical (IHC) staining. The tissue sections were blocked with 10% donkey serum for 1 h and incubated with primary antibodies against hnRNPA2B1or PCK1 (1:200, #A1162, #A22172, ABclonal, China) or Ki67 (1:1000, #ab15580, Abcam, UK), and then incubated with biotinylated anti-rabbit IgG secondary antibodies. Finally, the sections were visualized with a Vectastain ABC kit (Vector) and developed with 3,3′-diaminobenzidine (DAB) (Genetech, China). Tissue array chips containing HCC tissues and adjacent normal liver tissues were purchased from Servicbio (ZL-LivHCC961, China) and immunohistochemistry was performed with hnRNPA2B1. The staining score was calculated based on staining intensity and the percentage of positive cells.

### CCK-8 assay and colony formation assay

Cell proliferation was tested by CCK-8 kit (Dojondo, Japan) and the absorbance was measured at 450 nm at different time points (0, 24, 48, and 72 h) to determine the proliferation rate. Experiments were independently repeated three times. To evaluate the ability of colony formation of HCC cells, the cells were seeded at a low density of 1000 cells per well of 6-well plates and allowed to grow for approximately 14 days to form visible colonies. The cells were then fixed with 4% paraformaldehyde (PFA) about 15 min at room temperature and stained with 0.2% crystal violet (Solarbio, China) for 30min and photographed.

### Transwell migration and invasion assay

Cell migration and invasion were determined by Transwell chambers with 8μm pore size (Corning, USA). As for migration assay, HCC cells (4 × 10^4^ cells) were seeded into the upper chamber of the transwell in serum-free DMEM, while DMEM containing 10% FBS was added to the lower chamber. 48 h later, non-migrating cells on the upper chamber were removed and migrated cells attached to the lower side of the membrane were fixed with 4% PFA for 15 min and stained with 0.2% crystal violet. The migration cells were visualized using an inverted microscope (Leica, Germany) and were counted. Prior to the start of the invasion assay the upper chamber of the transwell plates were coated with Matrigel (Matrigel diluted 1:8 with serum-free medium) at 37 ℃ for 2 h, then HCC cells were seeded into the upper chamber. The other steps were the same as for the migration assay.

### Xenograft mouse models

Five-week-old male BALB/c nude mice were purchased from Shanghai SLAC Co. Ltd (Shanghai, China) and used in subcutaneous tumor-formation experiments. The mice were randomly divided into two groups of six mice per group. Huh7 cells infected with lentivirus containing hnRNPA2B1-sgRNA or NT sgRNA were injected right-back of each mouse. And 1 × 10^6^ cells were injected into each mouse. 28 days after injection cells, the mice were sacrificed, and the tumors were isolated and photographed. All animals were approved by the Institutional Animal Care and Ethics Committee of Renji Hospital, with a maximum allowable tumor volume of 2000 mm^3^.

### Hydrodynamic injection HCC models

We first constructed sgRNA plasmids targeting mouse hnRNPA2B1 and PCK1 genes based on the CRISPR-Cas9 method. The primers used to construct sgRNA plasmids are listed in Additional file 1: Table S2. The sgRNA plasmids were confirmed by sequencing. Next, six-week-old female C57BL/6 mice were randomly grouped into six mice per group. 2 ml plasmid mixture in 0.9% sodium chloride solution was prepared for each mouse. The plasmid mixture contained 13 μg pT3-EF1A- MYC-IRES-luc (Addgene 129775) (22), 6.5 μg pX330-p53 (Addgene 59910) (23), and 6.5 μg CMV-SB13 transposase, along with either hnRNPA2B1-sgRNA/PCK1-sgRNA or NT sgRNA plasmid. Then, 2 ml plasmid mixture was injected into the tail vein within 5–7 s. Mice were monitored by abdominal palpation and euthanized when they had a high burden of liver tumors.

### RNA sequencing

First, we harvested hnRNPA2B1 sgRNA Huh7 cells and NT sgRNA Huh7 cells and total RNA was extracted using an RNA Isolation Kit (OMEGA, Norcross, USA). The purity and quality of total RNA were assessed by Nanodrop and Agilent 2100 Bioanalyzer. dsDNA library was constructed using VAHTS Universal V6 RNA-seq Library Prep Kit (Vazyme, China) and was purified using VIHTS DNA Clean Beads (Vazyme, China). Then, the dsDNA library was qualified and quantified by Qsep-400 and Qubit TM dsDNA Assay kit. The qualified dsDNA samples were subsequently sequenced using the Illumina Nova Seq 6000 platform (San Diego, USA). Raw sequencing reads were mapped to GRCh38 assembly of the human genome using Tophat2, version 2.0.10 [21]. Fragments Per Kilobase of transcript per Million mapped (FPKM) were computed using Stringtie and normalized with trimmed mean of M values (TMM) [22, 23]. Differentially expressed genes (DEGs) were analyzed using DESeq 2. Genes with log-fold change > 1.5 and false discovery rate (FDR) < 0.05 were considered significantly transcriptomic changes. This sequencing dataset is available at GEO: GSE226544.

### Metabolite analysis

When cells were cultured up to 90% confluency, the cells were collected with a cell scraper after washed with PBS, and the cells were pelleted by centrifuging and stored in liquid nitrogen. A homogenate of 50 mg of sample mixed with 1 mL of cold methanol/acetonitrile/H2O (2:2:1, v/v/v) was sonicated twice (30 min/ once) at a low temperature and then incubated at − 20 ℃ for 1 h, followed by centrifugation for 20 min (140,00*g*, 4 °C). The supernatant was dried in a vacuum centrifuge. Sextuplicate samples were collected and sent to analyze metabolites by Metabolon-associated energy metabolism (Applied Protein Technology, Shanghai, China). For LC–MS analysis, the dried samples were dissolved in 100 μL acetonitrile/H2O (1:1, v/v). The samples were separated by Agilent 1290 Infinity LC ULTRA performance liquid chromatography system. Then A 5500 QTRAP mass spectrometer (MS, AB SCIEX) was used to analyze the chromatographic peak area and retention time in anion mode.

### RNA binding protein immunoprecipitation (RIP) assay

For the RIP experiment, 1 × 10^7^ cells were lysed in RIPA buffer (50 mM Tris–HCl, pH 8.0, 150 mM NaCl, 5 mM EDTA, 1% NP-40) for 10 min. After centrifugation, the cell lysates were pre-cleared with Protein A/G beads (Santa Cruz, Shanghai, China) and then incubated for 2 h with the antibody against hnRNPA2B1 or IgG. After washing, proteins and DNA were digested with proteinase K and DNase I. Total RNAs were extracted using TRIzol reagent and RT-PCR analysis was then performed on the purified RNA.

### Methylated RNA immunoprecipitation (MeRIP) assay

MeRIP assay was performed with a riboMeRIP m6A transcriptome Profiling Kit (RIBOBIO, China) according to the guidelines of the manufacturer. Briefly, First, RNA fragmentation. The total RNA of HCC cells was first extracted using TRIzol reagent (Thermo Fisher Scientific, USA), and then 18 μγ RNA at 1 μγ/ml was fragmented in the RNA fragmentation Buffer according to the procedures of the manufacturer. Next, fragmented RNAs were precipitated with ethanol. Second, Preparation of the m6A magnetic beads. Protein A/G magnetic beads were incubated with m6A antibody for 30 min, then washing for 3 times. Third, Immunoprecipitation. Magnetic beads with m6A antibody were incubated with the above fragmented RNAs in IP buffer for 2 h at 4 ℃. Then, the magnetic beads with m6A Ab and RNAs were added into the pipes adsorbed on the magnetic frames and washed. After that, m6A methylated RNAs were eluted and purified. Fourth. RT-PCR according to described above. The primer of PCK1 was listed in Additional file 1: Table S3.

### Immune infiltration analysis

ImmuCellAI (http://bioinfo.life.hust.edu.cn/web/ImmuCellAI) was used to predict the infiltration abundance of 36 immune cell types in cancer tissue using RNA-Seq data or gene-expression profiles derived from microarray data [24]. The normalized gene expression matrix was uploaded to ImmuCellAI for immune infiltration analysis. The Wilcoxon rank sum test to compare difference between groups. Spearman correlation analysis was performed to investigate the correlation of the expression of hnRNPA2B1 and immune cells.

### Statistical analysis

Statistical analysis of bioinformatic analysis was performed by R software version 4.1.3. At least three independent tests were performed for each cell culture experiment and the data are presented as mean ± SEM and the statistical analysis was evaluated by SPSS software 22.0 and statistical significance was assessed using the Student t-test to compare two groups, and the Chi-square test was used for the comparisons of categorical variables. Pearson correlation analysis was employed to determine the association between hnRNPA2B1 and other genes. Kaplan–Meier survival analysis was conducted to investigate the impact of hnRNPA2B1 on overall survival (OS) and relapse-free survival (RFS) in patients with liver cancer. The log-rank test was used to determine the statistical significance. p-values less than 0.05 indicates statistical significance. *p <0.05, **p <0.01, ***p <0.001, ****p <0.0001.

## Results

### hnRNPA2B1 is highly expressed in HCC and associated with a poor prognosis in HCC

**Fig. 1:**
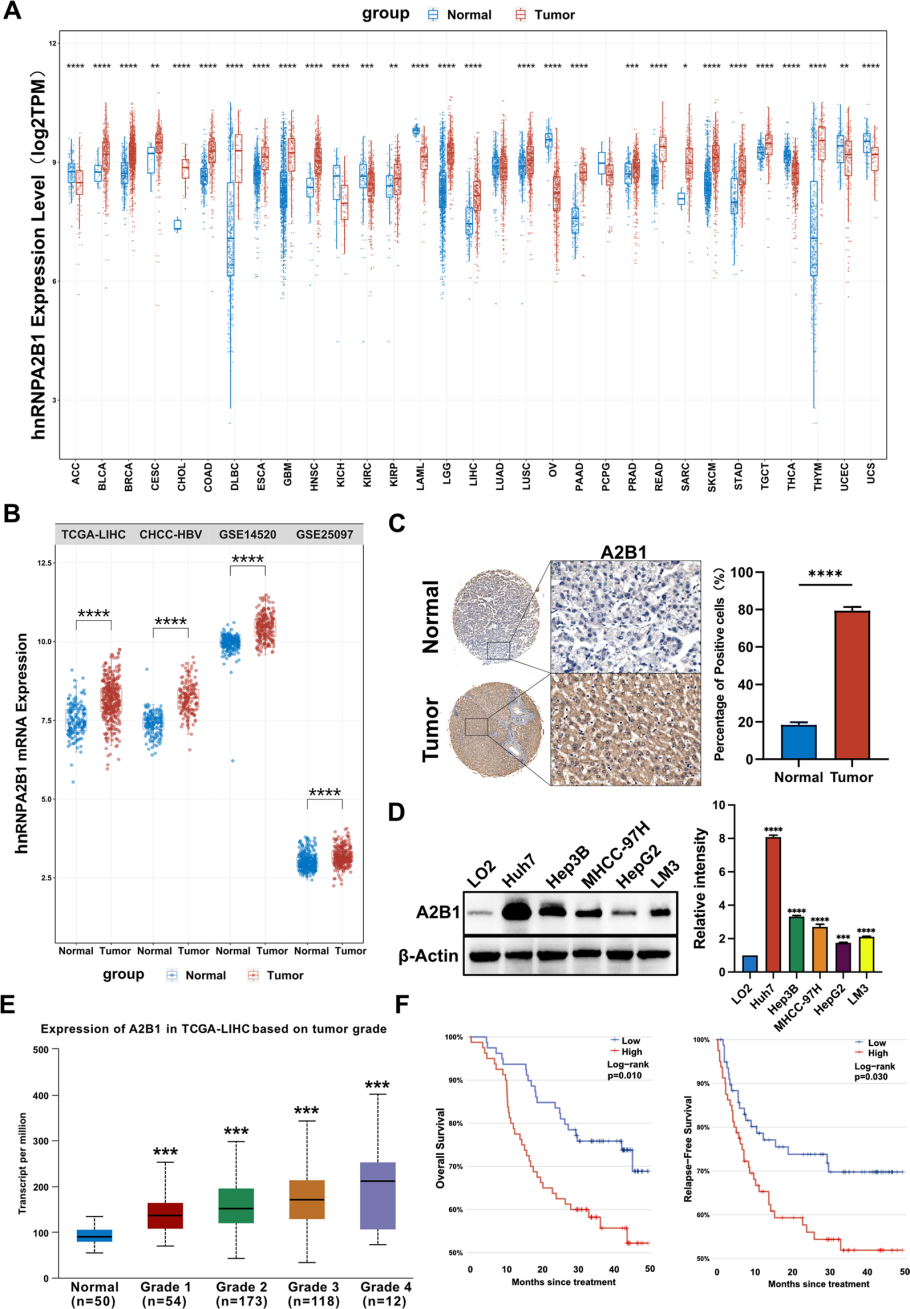
hnRNPA2B1 is highly expressed in HCC and its higher expression is associated with advanced clinical stages and a poor prognosis. **A** Analysis of hnRNPA2B1 mRNA expression levels in TCGA database. *p < 0.05, **p < 0.01, ***p < 0.001, ****p < 0.001 compared to normal tissues. **B** Analysis of hnRNPA2B1 mRNA expression levels in TCGA-LIHC, CHCC-HBV, GSE14520, and GSE25097 databases. **C** Representative images of immunohistochemistry staining of hnRNPA2B1 in HCC and adjacent non-tumor tissues from different HCC patients (left) and quantitative analysis of sample numbers at different hnRNPA2B1expression levels in 48 pairs of HCC tissues (right). Tumor indicates HCC tissue, Normal indicates adjacent normal tissue. **D** Western blot analysis of hnRNPA2B1 protein levels in non-tumorigenic LO2 liver cells, hepatocellular carcinoma cell lines Huh7, Hep3B, MHCC-97H, HepG2, and LM3 (left) and quantification of relative intensity of hnRNPA2B1 protein levels (right). A2B1 represents hnRNPA2B1. The levels of β-actin were used as an internal control. ***p < 0.001, ****p < 0.001. **E** The relationship between A2B1 mRNA levels and clinical stage was analyzed using data from TCGA databases. Statistical analysis was performed using one-way ANOVA to determine the significance of differences by comparing all groups to the "normal" tissue. ***p < 0.001, ****p < 0.001. **F** Overall survival and relapse-free survival in TCGA-LIHC database

To investigate whether hnRNPA2B1 has a tumorigenic function, we analyzed the TCGA database to study the expression of hnRNPA2B1 in human various tumors. We found that hnRNPA2B1 was highly expressed in various tumors, especially in HCC (also named LIHC) (Fig. 1A). We also confirmed the high expression of hnRNPA2B1 in HCC using other HCC databases from CHCC-HBV, GSE14520, and GAS25097 (Fig. 1B). Moreover, the tissue microarray of 48 pairs of HCC tissues from patients showed that the expression of hnRNPA2B1 was more strongly expressed in tumor tissues of HCC higher than that in adjacent non-tumor tissues (Fig. 1C, Additional file 1: Fig. S1). In addition, we determined the expression level of hnRNPA2B1 in HCC cell lines using western blot and found that hnRNPA2B1 was expressed at a higher level in Huh7, Hep3B, MHCC-97H than non-tumorigenic LO2 liver cells (Fig. 1D). Overall, these results demonstrate that hnRNPA2B1 is upregulated in HCC.

As the occurrence and progression of HCC are closely related to the viruses HBV and HCV [25], we analyzed the correlation of hnRNPA2B1 RNA level with HCC patients with HBV/HCV infection based on a TCGA -LIHC database. Figure S2-A shows that the expression of hnRNPA2B1 expression was not correlation with HCC patients with or without HBV infection. In addition, we found that the expression of hnRNPA2B1 was higher in patients co-infected with HBV and HCV, but not in patients without HBV infection or only with HBV or HCV infection (Additional file 1: Fig. S2B). The results indicate that the expression of hnRNPA2B1 is associated with HBV and HCV co-infection. Next, we further analyzed the correlation of hnRNPA2B1 RNA level with clinical malignant grades of HCC based on the TCGA-LIHC database. As shown in Fig. 1E, the high expression of hnRNPA2B1 was correlated to the advanced clinical stage for HCC patients. Moreover, we also analyzed the correlation of hnRNPA2B1 RNA level, with patients at different clinical malignant grades, HBV/HCV infection, or age. Additional file 1: Fig. S2C shows that the high expression of hnRNPA2B1 was only correlated with these patients advanced clinical grade without HBV infection or with HBV and HCV co-infection, but not with these patients advanced clinical grade with HBV or HCV infection. Besides, Figure S2D shows that the high expression of hnRNPA2B1 was only correlated with these patients advanced aged 35–65 years with advanced clinical grade. Additionally, HCC patients with higher hnRNPA2B1 had poor overall survival and relapse-free survival (Fig. 1F). Collectively, the results indicate that the higher expression of hnRNPA2B1 is correlated to a more advanced clinical stage and a poorer prognosis of HCC patients, as well as HBV and HCV co-infection.

### Knockout of hnRNPA2B1 inhibits cell proliferation, migration, and invasion in HCC cells

**Fig. 2:**
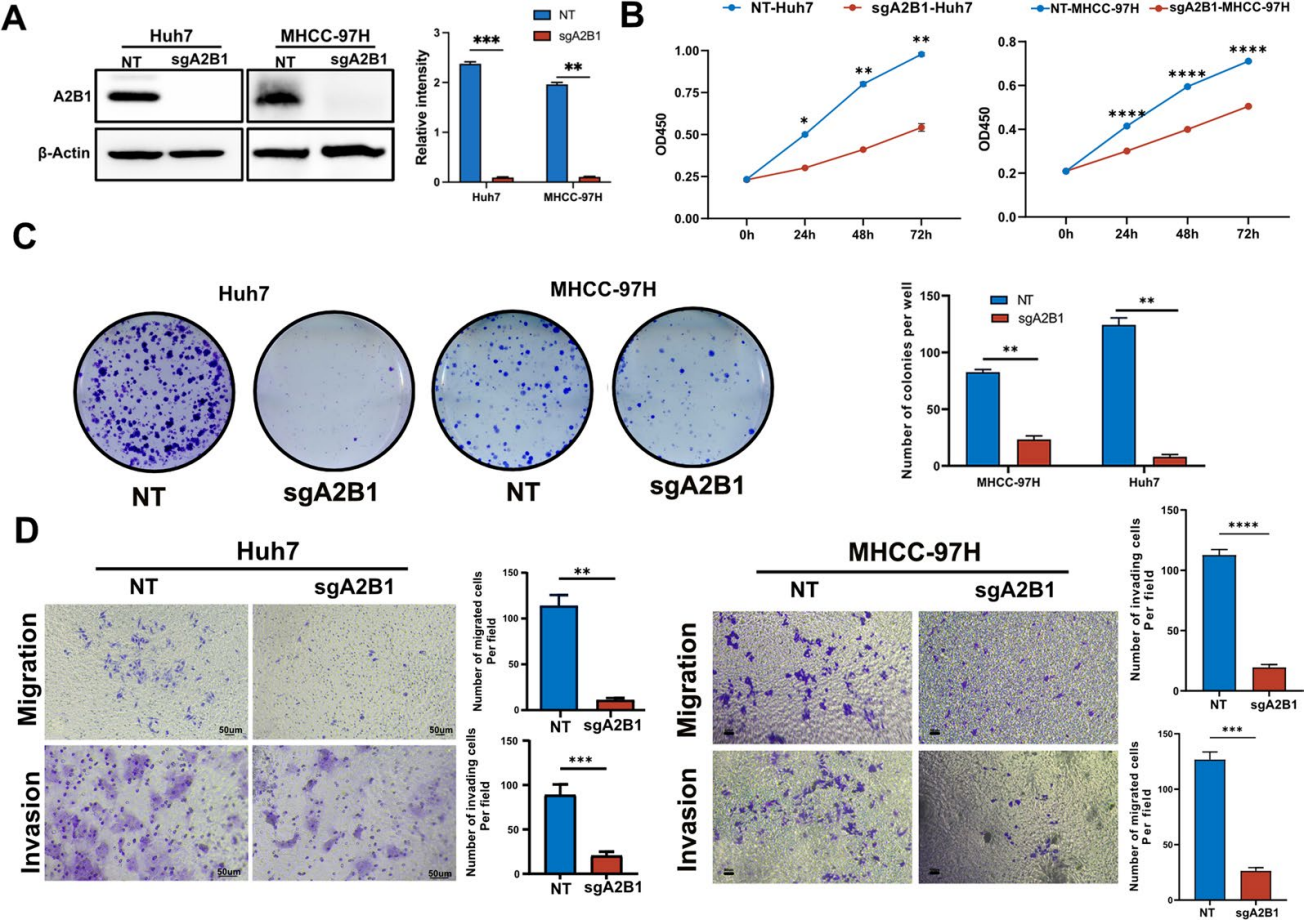
Knockout of hnRNPA2B1 (A2B1) inhibits the proliferation, migration, and invasion of HCC cells in vitro. **A** Knockout of A2B1 in Huh7 and MHCC-97H cells were determined by western blot (left) and quantification of the relative intensity of A2B1 protein levels (right). The levels of β-actin were used as an internal control. **B** The cell proliferation ability of A2B1 knockout Huh7 and MHCC-97H cells was measured by CCK-8 assay. **C** Colony-forming assay of A2B1 knockout Huh7 cells and MHCC-97H cells (left) and quantification of the number of colonies in these cells (right). **D** The migration and invasion capacity was evaluated in sgA2B1-Huh7 cells and sgA2B1-MHCC-97H cells by the transwell assay and quantification of migrated cells and invading cells per field. At least six fields were counted in every group. sgA2B1 represents hnRNPA2B1-sgRNA. NT represents non-sense sgRNA used as a control of A2B1-sgRNA. *p < 0.05, **p < 0.01, ***p < 0.001, ****p < 0.0001, compared to NT control

To investigate the function of hnRNPA2B1 on HCC, we used a CRISPER/Cas 9 sgRNA strategy to knockout hnRNPA2B1 in HCC cell line Huh7 and MHCC-97 cells. Western blotting analysis showed that the expression of hnRNPA2B1 (also named A2B1) in A2B1-sgRNA Huh7 and MHCC-97H cells is much lower than that in the non-sense sgRNA (NT) control cells (Fig. 2A). As sown in Fig. 2B, knockout of A2B1 remarkably inhibited the cell proliferation of Huh7 and MHCC-97H cells based on CCK8 assay. Moreover, the colony-formation capacity of A2B1-sgRNA Huh7 and MHCC-97H cells was much weaker than the NT control group under low-density culture conditions (Fig. 2C). Subsequently, we performed a transwell assay and found that knockout of A2B1 dramatically restrained the migration and invasion capacity of Huh7 cells and MHCC-97H cells (Fig. 2D). Taken together, these results indicate that the knockout of A2B1 inhibits cell proliferation, migration, and invasion of HCC cells.

### Overexpression of A2B1 promotes proliferation, migration and invasion in HCC cells

**Fig. 3:**
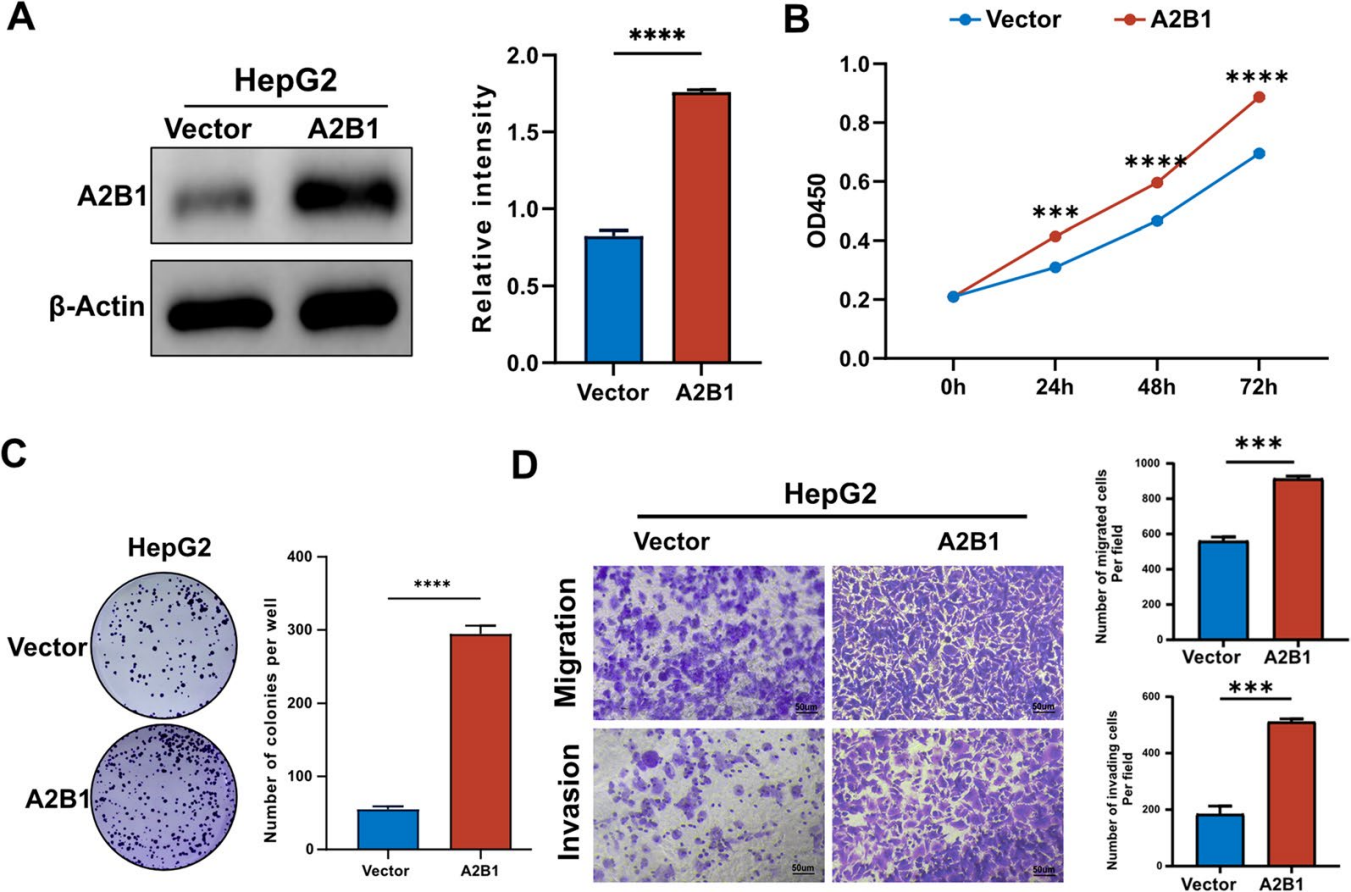
Overexpression of A2B1 promotes the cell proliferation, migration, and invasion of HCC cells in vitro. **A** The stable overexpression of A2B1 in HepG2 cells was determined by western blot (left) and quantification of the relative intensity of A2B1 protein levels (right). **B** The cell proliferation rate of overexpression of A2B1 HepG2 cells was measured by CCK8 assay. **C** Colony-forming assay of overexpression of A2B1 HepG2 cells (left) and quantification of the number of colonies formed (right). **D** The migration and invasion were measured in A2B1-overexpression HepG2 cells by transwell assay (left) and quantification of migrated cells and invading cells per field (right). A2B1 represents overexpression of A2B1. An empty vector was used as a control. Statistical analysis was performed using the student's T-test to determine the significance of differences. Scale bar, 50 μm. **p  < 0.001, ****< *p *0.0001

To further investigate the tumor-promoting effect of A2B1 on HCCs, we ectopically expressed A2B1 in HepG2 cells. Western blotting verified that the expression level of A2B1 in the overexpression group was much higher than that in the vector control (Fig. 3A). As shown in Fig. 3B, overexpression of A2B1 significantly promoted the cell proliferation of HepG2 according to CCK-8 assay. We then performed colony-forming assays of A2B1 and found that overexpression of A2B1 increased the total number of colonies compared to the vector control (Fig. 3C). In addition, overexpression of A2B1 enhanced the cell migration and invasion potential of HepG2 (Fig. 3D). Overall, these data imply that overexpression of A2B1 promotes cell proliferation, migration, and invasion in HCC lines.

### A2B1 promotes tumorigenesis and procession of HCC in vivo

**Fig. 4:**
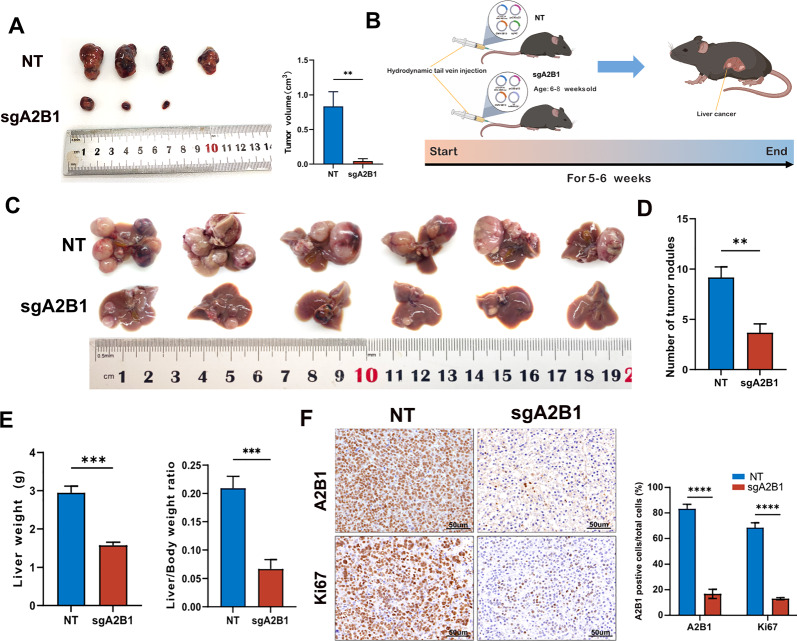
Knockout of A2B1 inhibits the tumorigenesis and progression of HCC. **A** In vivo subcutaneous tumor-formation experiments of A2B1 sgRNA Huh7 and NT control Huh7 cells. **B** A schematic illustration of the strategy of the hydrodynamic tail vein injection HCC model. A mixture of plasmids pT3-EF1A-MYC-IRES-luc, px330-p53, and CMV-SB13 transposase, was delivered together with either A2B1 sgRNA or NT control for HCC induction. **C** Images of dissected livers in the A2B1 sgRNA and NT control group. **D** Quantification of the number of tumor nodules per mouse in the A2B1 sgRNA group or NT control group. **E** Quantification of the liver weight and the ratio of liver weight to body weight of these tumor nodules per mouse in A2B1 sgRNA or control groups. At least 6 mice were used in every group. **F** Representative images of immunohistochemical staining of the formed tumors in the liver tissues using A2B1 and Ki67 antibodies (left) and quantification of the percentages of A2B1 positive cells or Ki67 positive cells in total cells. At least 6 fields were counted for quantification analysis and at least 3 mice were used in every group. Scale bar, 50 μm. A. **p < 0.01, ***p < 0.001, and ****p  < 0.0001 compared to NT control

To explore if A2B1 can promote tumorigenesis and processing in HCC in vivo*,* we first performed subcutaneous tumor-formation experiments in nude mice. As expected, knockout of A2B1 in Huh7 cells significantly inhibited tumor formation in nude mice (Fig. 4A), indicating that high expression of A2B1 promoted tumor formation in vivo*.* To further validate the tumorigenesis-promoting function of A2B1, we knocked out endogenous A2B1 by mouse A2B1 sgRNA in an immunocompetent mouse model of orthotopic tumorigenesis in the liver using the hydrodynamic tail vein injection delivery protocol described previously [26]. In brief, we simultaneously injected four plasmids including pT3-EF1A-Myc-IRES-Luc, transposase CMV-SB13, P53 sgRNA px330-P53, and sgA2B1 or NT into the mouse tail vein within 5–7s (Fig. 4B). After 35–40 days post-injection, tumors nodules formed in the liver tissue. As shown in Fig. 4C–E, knockout of A2B1 significantly reduced the size and number of tumor nodules, and liver weight compared to the NT control group. Moreover, western blot and immunohistochemical analysis using A2B1 antibody demonstrated that the expression levels of A2B1 in the tumor nodules were dramatically lower than that in the NT control group. The same phenomenon was observed for Ki67 staining (Fig. 4F). These findings indicate that A2B1 plays a key role in triggering tumorigenesis and mediating tumor progression in vivo*.*

### A2B1 is negatively associated with the gluconeogenic pathway

**Fig. 5:**
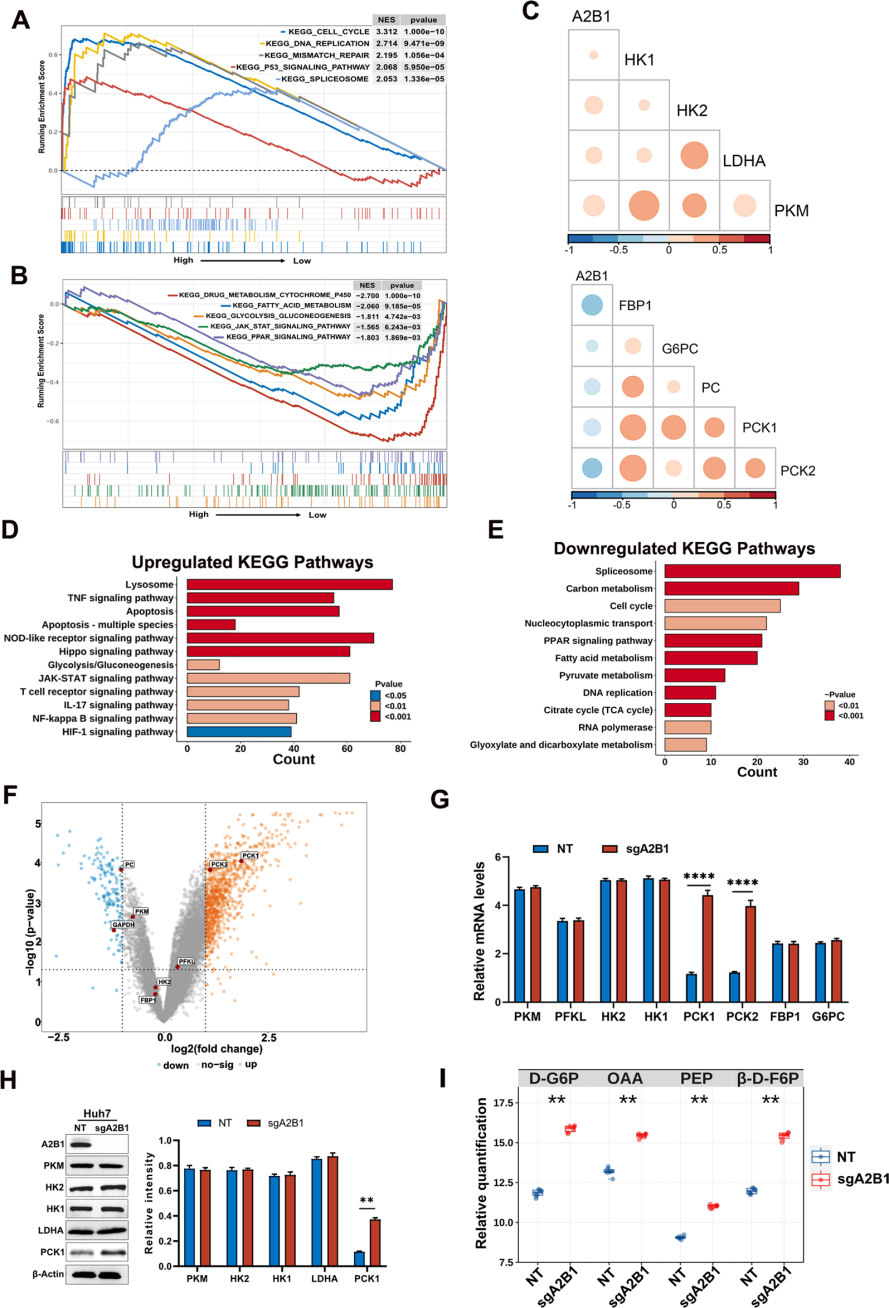
Functional enrichment analysis of A2B1 and correlation analysis of A2B1 and glycolysis/gluconeogenic pathway. **A**, **B** GSEA analysis of top upregulated pathways (**A**) and downregulated pathways (**B**) of A2B1-related HCCs based on the TCGA-LITC database. **C** The correlation analysis of A2B1 expression of glycolysis/gluconeogenic pathways. **D**, **E** KEGG pathway enrichment analysis of upregulated and downregulated pathways in sgA2B1 Huh7 cells versus NT Huh7 cells. **F** Volcano plot of expressed gene expression of NT Huh7 cells and sgA2B1 Huh7 cells. Key rate-limiting enzymes related to glycolysis/gluconeogenic pathways were marked. **G** RT-PCR analysis of rate-limiting enzymes related to glycolysis/gluconeogenic pathways. **H** Western blot analysis of glycolysis-related enzymes and PCK1 (left) and quantification of the relative intensity of these protein levels (right). **I** Relative quantification of key metabolites of gluconeogenesis in NT Huh7 cells and sgA2B1 Huh7 cells (n = 6)

To investigate the molecular mechanisms, we divided the HCC patients into two groups (high expression of the A2B1 group and low expression of the A2B1 group) from the TCGA-LIHC database. Through GSEA enrichment analysis, we found that the top upregulated pathways in high expression of the A2B1 group were correlated with cell cycle, DNA replication, mismatch repair, spliceosome, and P53 signaling, which are involved in cell proliferation (Fig. 5A). This analysis also revealed a correlation between A2B1 and DNA replication, transcription, and mRNA splicing, whereas the downregulated pathways were associated with metabolism and metabolism-related pathways, including drug metabolism, glycolysis/gluconeogenesis, lipid acid metabolism, and PPAR signaling pathway[27] (Fig. 5B). Aberrant lipid metabolism had been investigated as a diagnostic and therapeutic target in liver cancer [28, 29]. In the present study, we focused on the glycolysis/gluconeogenesis pathway. Correlation analysis showed that glycolysis related rate-limiting enzymes hexokinase 1 (HK1), HK2, L-lactate dehydrogenase A(LDHA), and pyruvate kinase (PKM) were positively correlated with the expression level of A2B1 in HCC. However, gluconeogenesis related rate-limiting enzymes Fructose-Bisphosphatase 1 (FBP1), Glucose-6-Phosphatase Catalytic Subunit 1 (G6PC), PC (pyruvate carboxylase), phosphoenolpyruvate carboxykinase 1 (PCK1), and PCK2 were negatively correlated with the expression level of hnRNPA2B1 in HCC (Fig. 5C).

Next, to further confirm the regulated genes and pathways related to A2B1, we performed RNA sequencing analysis with A2B1 sgRNA and NT control in Huh7 cells. Heat map displayed the upregulated genes and downregulated genes in A2B1 sgRNA groups (Additional file 1: Fig. S3). KEGG pathway enrichment analysis showed that the top upregulated pathways in A2B1 sgRNA groups were related to the lysosome, apoptosis, glycolysis/gluconeogenesis, and inflammation-related pathways (Fig. 5D), indicating that knockout of A2B1 promotes cell apoptosis and inflammation. On the contrary, knockout of A2B1 downregulates cell proliferation-related pathways, including cell cycle, DNA replication, RNA metabolism; metabolism-related pathways, such as carbon metabolism, fatty acid metabolism, PPAR signaling, tricarboxylic acid (TCA) cycle (Fig. 5E), suggesting that knockout of A2B1 inhibits cell proliferation and metabolism. Here, we emphasized on the glycolysis/gluconeogenesis pathway related to A2B1. Volcano plot shows that the expression glycolysis-related enzymes PKM, phosphofructokinase, Liver Type (PFKL), HK1 and HK2 were unchanged in A2B1 sgRNA Huh7 cells, while the gluconeogenesis-related rate-limiting enzymes PCK1 and PCK2 were upregulated (Fig. 5F). Next, we further measured the expression of glycolysis-related enzymes and gluconeogenesis-related enzymes by RT-PCR and western blot and confirmed that PCK1 and PCK2 were upregulated in A2B1 sgRNA groups (Fig. 5G, H). Gluconeogenesis is the synthesis of glucose from non-carbohydrate precursors and can antagonize aerobic glycolysis in cancer via the gluconeogenesis-related key enzymes PCK1/PCK2, FBP, and G6PC [30]. PCK1 is a cytosolic isoform of PCK and it is downregulated in HCC. Therefore, A2B1 might downregulate gluconeogenesis mainly via PCK1.

To confirm that A2B1 downregulates gluconeogenesis, we further measured the intracellular metabolites of gluconeogenesis in NT and A2B1 sgRNA HuH7 cells using high-performance liquid chromatography/mass spectrometry (HPLC/MS) and gas chromatography/MS (GC/MS). We found that metabolites D-Glucose 6-phosphate (D-G6P), Oxaloacetic acid (OAA), Phosphoenolpyruvic acid (PEP), B-D-Fructose 1,6-bisphosphate (β-D-F6P) in gluconeogenesis process was upregulated A2B1 sgRNA groups (Additional file 1: Table S4, Fig. 5I). In summary, the above results suggested that A2B1 downregulate gluconeogenesis via PCK1. Therefore, we next focused on the molecular mechanism of A2B1 regulating PCK1 on HCC.

### A2B1 negatively regulates the expression of PCK1 gene by m6A methylation

**Fig. 6:**
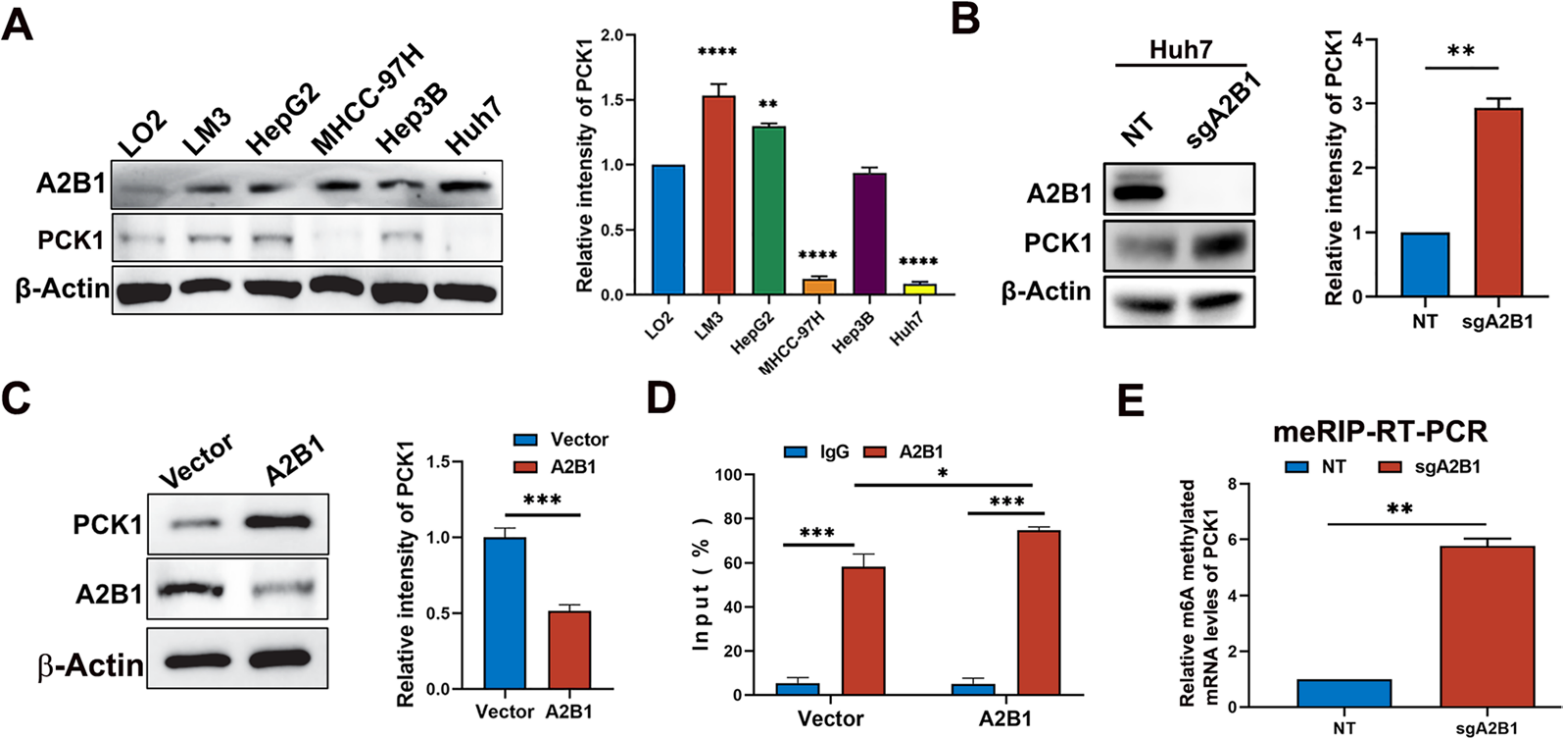
A2B1 negatively regulates the expression level of PCK1 by m6A modification. **A** Western blot analysis of the protein levels of A2B1 and PCK1 in LO2 liver cells, HCC cell line LM3, HepG2, MHCC-97H, Hep3B, and Huh7 (left) and quantification of relative intensity of PCK1 protein levels (right). **B** Western blot analysis of the protein levels of A2B1 and PCK1 in NT Huh7 and sgA2B1 Huh7 cells (left) and quantification of relative intensity of PCK1 protein levels (right). **C** Western blot analysis of the protein levels of A2B1 and PCK1 in Vector HepG2 cells and A2B1 overexpressed HepG2 cells (left) and quantification of the relative intensity of PCK1 protein levels (right). **D** RIP analysis of relative PCK1 mRNA levels bound to A2B1 proteins using A2B1 antibody in vector HepG2 cells and A2B1 overexpressed HepG2 cells. IgG was used as a control of the A2B1 antibody. **E** MeRIP analysis of relative m6A methylated mRNA levels of PCK1 using m6A antibodies in NT Huh7 cells and sgA2B1 HuH7 cells. Statistical analysis was performed using one-way ANOVA to determine the significance of differences. *p  < 0.05, **p < 0.01, ***p < 0.001, ****p  < 0.001

Next, we further investigated whether A2B1 affects the expression of PCK1. We first determined the expression levels of PCK1 and A2B1 in HCC cell lines by western blot and found that the expression of PCK1 was low (Fig. 6A). Moreover, the expression of A2B1 was higher in MHCC-97H and Huh7 cells, the lower levels of PCK1 were observed in the two HCC cells (Fig. 6A). Furthermore, knockout of A2B1 in Huh7 cells enhanced the protein expression of PCK1 (Fig. 6B), while, overexpression of A2B1 in HepG2 cell decreased the protein expression of PCK1 (Fig. 6C). Therefore, these results indicate that A2B1 negatively regulates the expression of PCK1. Next, we wondered if A2B1 bound the PCK1 mRNA. Thus, we performed an RIP experiment using an A2B1 antibody in HepG2 cells. As shown in Fig. 6D, A2B1 bound more mRNAs of PCK1 than IgG. Moreover, overexpression of A2B1 could pull down more PCK1 mRNAs than the vector control group. The data implies that A2B1 can bind the PCK1 mRNAs. Subsequently, we investigated if regulating the mRNA levels of PCK1 by A2B1 was dependent on the m6A mRNA modification We performed MeRIP with NT HuH7 cell and sgA2B1-HuH7 cells and found that m6A-methyl mRNA levels of PCK1 were significantly higher in sgA2B1 HuH7 cells than that in NT HuH7 cells (Fig. 6E). The results indicated that A2B1 downregulates the mRNA levels of PCK1 by m6A methylataion.

### The tumor growth and progression promoting of A2B1 in HCC is dependent on the lower expression level of PCK1

**Fig. 7:**
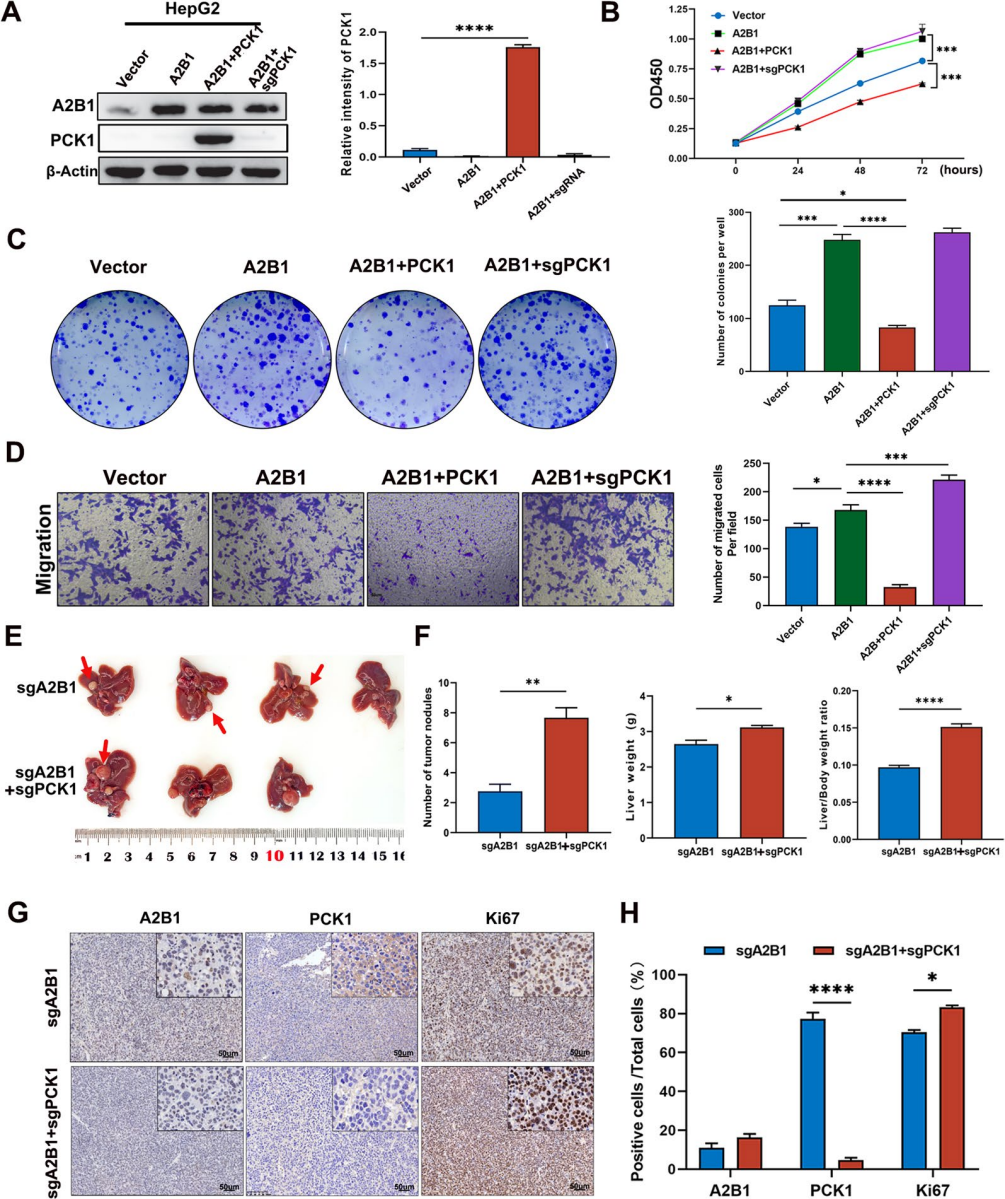
The tumorigenesis and progression-promoting effects of A2B1 in HCC are dependent on the expression level of PCK1. **A** Overexpression or knockout of PCK1 in A2B1-overexpressing HepG2 cells was determined by western blot (left) and quantification of the relative intensity of PCK1 protein level (right). **B** The cell proliferation rate of overexpression or knockout of PCK1 in A2B1-overexpressing HepG2 cells was measured by CCK8 assay. **C** Colony-forming assay of overexpression or knockout of PCK1 in A2B1-overexpressing HepG2 cells (left) and quantification of the number of colonies formed per well (right). **D** The cell migration was measured in overexpression or knockout of PCK1 in A2B1 overexpressed HepG2 cells (left) and quantification of migrated cells per field (right). A2B1 and PCK1 represent overexpression of A2B1 and PCK1, respectively. An empty vector was used as a control. **E** Images of dissected livers in the A2B1 sgRNA group and the A2B1/PCK1 double sgRNA group, respectively. sgA2B1 represents A2B1 sgRNA, and sgPCK1 represents PCK1 sgRNA. **F** Quantification of the number of tumor nodules per mouse and the liver weight and the ratio of liver weight in the A2B1 sgRNA group or sgA2B1 and sgPCK1 group. At least 6 mice were used in every group. **G**, **H** Representative images of immunohistochemical staining of the formed tumors in the liver tissues using A2B1, PCK1, and Ki67 antibodies (left) and quantification of the percentages of A2B1 or PCK1 or Ki67 positive cells in total cells (right)

Our above-described data have demonstrated that A2B1 promotes the occurrence and progression of HCC and A2B1 regulates PCK1 expression (Figs. 4, 6). Next, we wonder whether the tumorigenic function of A2B1 on HCC is dependent on the expression of PCK1. First, we knockout of PCK1 or overexpressed PCK1 in A2B1 overexpressing HepG2 cells. Western blot analysis showed that the level of PCK1 was low when A2B1 was overexpressed, however, the level of PCK1 was restored when PCK1 was overexpressed in A2B1 overexpressing HepG2 cells, while sgPCK1 had no such effect (Fig. 7A). Next, we measured the cell proliferation of HepG2 cells in different groups and found that overexpression of PCK1 significantly inhibited the proliferating-promoting effect of A2B1 in HepG2 induced by the high levels of A2B1, even the proliferation rate of HepG2 cells in the group was lower than that of vector control (Fig. 7B). However, knockout of PCK1 abrogate the effect of PCK1 in A2B1-overexpressing HepG2 cells (Fig. 7B). The similar effects of PCK1 on the clone formation and cell migration of HepG2 cells were observed when A2B1 was overexpressed (Fig. 7C, D). These results indicate that the promoting effect of A2B1 on the proliferation, clone formation, and migration is dependent on the lower expression of PCK1. Considering our above-described data demonstrating that knockout of A2B1 evidently suppressed the tumorigenesis and progression of HCC in vivo (Fig. 4), we further validate whether the function of A2B1 on the tumorigenesis and progression of HCC in vivo is dependent on the low expression of PCK1. We applied an in vivo spontaneous tumor model above described. As shown in Fig. 7E, F, knockout of PCK1 partly countered the tumor-inhibiting effect of knockout of A2B1 on HCC*.* Immunohistochemical staining confirmed that the expression level of PCK1 was lower in the tumor tissues formed in A2B1 and PCK1 double knockout mice than A2B1 sgRNA group. Moreover, the expression level of Ki67 in these formed tumor tissues was higher than that in A2B1 sgRNA groups (Fig. 7G, H). The above results imply that the tumorigenesis and progression-promoting effects of A2B1 are dependent on the lower expression of PCK1.

### Correlation between A2B1 level and immune infiltration in HCC

Considering that immune infiltration cells play a vital role in tumor microenvironment of HCC [31], we assessed the correlation between A2B1 expression and immune cell infiltration in CHCC cohort. Heat map shows that expression of A2B1 is weakly associated the immune cells infiltration, such as CD4 + T cell, Treg cells, DC, NK, Neutrolphil and monocytes, MAIT, CD8 + T cells, Exhausted immune cells (Additional file 1: Fig. S4A, S4C). We further perform Spearman's correlation tests to investigate the correlation between hnRNPA2B1 and immune features, we found that hnRNPA2B1 expression was weakly positively correlated with CD8 + T, Exhausted and DC cell infiltration (Additional file 1: Fig. S4B). The results for the TCGA database are similar to the former (Additional file 1: Fig. S5). Therefore, the results revealed that the expression of A2B1 is weakly correlation with immune infiltration in HCC.

## Discussion

m6A modification plays an important role in tumor initiation and progression [15]. m6A RNA methylation modulators (“writer”, “reader” and “erase” proteins) were found to be significantly different between HCC and normal tissues based on TCGA data analysis [11, 12]. In this study, we demonstrate that hnRNPA2B1 (named as A2B1), as a reader of m6 A RNA methylation, is highly expressed in HCC patients. More importantly, based on univariate and multivariate Cox regression analysis, we find that the high expression of A2B1 is correlated with advanced clinical stages and a poor prognosis. Therefore, A2B1 might be an independent prognostic risk factor for HCC. Moreover, we investigated that the function of A2B1 both in vitro and in vivo and found that A2B1 promotes the proliferation, migration, and invasion of HCC cells*.* Consistently*,* Huh7 cells transfected with A2B1 sgRNA inhibit tumor formation by a subcutaneous graft experiment and endogenous knockout of mouse A2B1 dramatically reduces the carcinogenic ability in an immunocompetent HCC mouse model. Taken together, these results demonstrate that A2B1 promotes tumorigenesis and progression of HCC.

As for the molecular mechanism of A2B1 on HCC, GSEA and KEGG enrichment analyses based on the GSAE-HCC database revealed that high expression of A2B1 is associated with cell cycle, DNA replication, and mismatch repair, which contribute to tumor proliferation [32]. Of note, GSEA analysis indicates that the gluconeogenesis process is downregulated in HCC patients expressing high levels of A2B1. The correlation analysis also reveals that gluconeogenesis is the negatively correlated with A2B1. Recent studies have demonstrated that gluconeogenesis plays a balanced role in controlling aerobic glycolysis by cancer cells [30]. Gluconeogenesis, an essential metabolic process in hepatocytes, is reported downregulated in HCC [33, 34]. Our finding shows that gluconeogenesis-related key enzyme PCK1 was downregulated in cancer tissues of HCC than in normal tissues. Moreover, PCK1 is negatively correlated with A2B1 expression. In vitro function experiments showed that knockout of A2B1 dramatically increases the expression of PCK1, whereas overexpression of A2B1 significantly reduces the expression of PCK1. These results indicate that A2B1 downregulates PCK1 mRNA levels. As for the effect of PCK1 on the tumorigenic role of A2B1, overexpression of PCK1 in HepG2 cells reduces the proliferation, clone formation, and migration of HepG2 induced by overexpression of A2B1. Of note, endogenous knockout of PCK1 can partially antagonize the tumor inhibitory effects of A2B1 sgRNA in a HCC mouse model. Therefore, our results suggest that A2B1 promotes the initiation and progression of HCC by downregulating PCK1 and it is dependent on the low expression of PCK1.

A2B1 is a pre-mRNA-binding protein that participates in the regulation of pre-mRNA processing and mRNA stability [17]. Recent studies have demonstrated that A2B1 interacting with lncRNA MIR100HG stabilizes TCF7L2 mRNA in a m6A-dependent manner to promote colorectal cancer progression [20]. Jiang et al. reported that A2B1 recognizes the m6A sites of ILF3 and enhances the stability of ILF3 mRNA [19]. Our current study shows that high expression of A2B1 in HCC reduces m6A-containing PCK1 mRNA levels, however, knockout of A2B1 dramatically increases m6A methylated mRNA levels of PCK1, implying that A2B1 recognizes m6A sites of PCK1 and likely promotes the degradation of PCK1 mRNA. However, the mechanism of how A2B1 affects the stability of PCK1 mRNA is not fully elucidated. mRNA stability is controlled depending on specific cis-acting elements and trans-acting elements and their specific binding protein [35]. It is reported that A2B1 has different effects on the stabilization of mRNAs by binding to UAASUUUU sequences of 3′-UTR of human mRNAs. A2B1 specifically recognizes this motif and recruits the CCR4-NOT deadenylase complex to induce the deadenylation of mRNA, and therefore promotes mRNA decay [36]. Interestingly, we found that the 3′-UTR region contains the UAASUUAU sequence of PCK1 (data not shown), however, whether A2B1 recognizes the motif of PCK1 and it is dependent on m6A modification remains unclear. In addition, A2B1 can affect mRNA stability through alternative splicing. For example, the Na(v) 1,6 sodium channel transcript has two types of exon 18 (exon 18A and exon 18N). Exon 18N has a termination codon leading to nonsense-mediated mRNA decay [37]. A2B1 has been demonstrated to determine the selection of polyadenylation sites in the pre-mRNA processing of some genes, therefore affecting mRNA half-life [38]. Thus, more research is required in the future to understand the mechanism of A2B1 affecting the stability of PCK1 mRNA.

## Conclusions

In summary, our study reveals that the high expression of A2B1 is associated with a poor prognosis in HCC patients. More importantly, the high expression of A2B1 promotes the occurrences and progression of HCC by decreasing PCK1 mRNA levels via m6A modification. Therefore, this study indicates that A2B1 might be a novel diagnostic marker for prognosis and a molecular therapeutic target for HCC patients.

### Supplementary Information

**Additional file 1: Figure S1.** Representative immunohistochemistry images of hnRNPA2B1 expression in tissue microarrays of different HCC patients. T represents tumor tissues; N represents paired adjective non-tumor tissues. **Figure S2.** A. Differential expression of hnRNPA2B1 in HBV-positive and negative HCC patients. B. Differential expression of hnRNPA2B1 in HCC patients infected with HBV or HCV. C. Differences in hnRNPA2B1 expression across clinical grades in HCC patients infected with HBV or HCV. D. Differential expression of hnRNPA2B1 in HCC patients with age-specific clinical classifications. Statistical analysis was performed using student's T-test to determine the significance of differences. *p < 0.05, **p < 0.01, ***p < 0.001, ****p < 0.0001. **Figure S3.** A. Immune cell infiltration of the CHCC cohort was analyzed using the ImmuCellAI method. The median value of hnRNPA2B1 expression was used to divide the high and low groups. B. Correlation between hnRNPA2B1 expression and immune cell infiltration. C. hnRNPA2B1 expression and MAIT, CD8_T, Exhausted immune infiltration differences. **Figure S4.** Heatmap of differential expression of Huh7 cells in NT and sgA2B1 groups. (n = 3). **Figure S5.** Heatmap of the correlation between A2B1 expression and immune infiltration in HCC patients. **Table S1.** The detail of gene expression profiles of hepatocellular carcinoma. **Table S2.** Sequences for quantitative real-time PCR, PCR and sgRNA. **Table S3.** Comparison of differences in clinical information between patients grouped by high and low hnRNPA2B1 expression in the CHCC-HBV dataset. **Table S4.** Relative Quantification of metabolites in NT and sgA2B1 HuH7 cells using high-performance liquid chromatography/mass spectrometry (HPLC/MS) and gas chromatography/MS (GC/MS).

## Data Availability

Published data analyzed in this study are available from TCGA (https://portal.gdc.cancer.gov/), GEO (https://www.ncbi.nlm.nih.gov/geo/), CHCC-HBV. All data generated and used in this study are available for anyone to utilize upon requested.

## Abbreviations

HCC: Hepatocellular carcinoma
HBV: Hepatitis B Virus
HCV: Hepatitis C Virus
TCGA: The Cancer Genome Atlas
GEO: Gene Expression Omnibus
GO: Gene ontology
KEGG: Kyoto Encyclopedia of Genes and Genomes
GSEA: Gene set enrichment analysis
KO: Knock out
OE: Overexpression
PCK1: Phosphoenolpyruvate carboxykinase 1
RIP: RNA immunoprecipitation
MeRIP: Methylated RNA immunoprecipitation
IHC: Immunohistochemistry
OS: Overall survival
LIHC: Liver hepatocellular carcinoma
m6A: N6-methyladenosine
