# Supplementary material for: hnRNPA2B1 promotes the occurrence and progression of hepatocellular carcinoma by downregulating PCK1 mRNA via a m6A RNA methylation manner
Source: J Transl Med. 2023 Nov 28;21:861. doi: 10.1186/s12967-023-04704-4 (PMC10683354; doi:10.1186/s12967-023-04704-4)
Supplement: Supplementary file 1 — Additional file 1: Figure S1. Representative immunohistochemistry images of hnRNPA2B1 expression in tissue microarrays of different HCC patients. T represents tumor tissues; N represents paired adjective non-tumor tissues. Figure S2. A. Differential expression of hnRNPA2B1 in HBV-positive and negative HCC patients. B. Differential expression of hnRNPA2B1 in HCC patients infected with HBV or HCV. C. Differences in hnRNPA2B1 expression across clinical grades in HCC patients infected with HBV or HCV. D. Differential expression of hnRNPA2B1 in HCC patients with age-specific clinical classifications. Statistical analysis was performed using student's T-test to determine the significance of differences. *p < 0.05, **p < 0.01, ***p < 0.001, ****p < 0.0001. Figure S3. A. Immune cell infiltration of the CHCC cohort was analyzed using the ImmuCellAI method. The median value of hnRNPA2B1 expression was used to divide the high and low groups. B. Correlation between hnRNPA2B1 expression and immune cell infiltration. C. hnRNPA2B1 expression and MAIT, CD8_T, Exhausted immune infiltration differences. Figure S4. Heatmap of differential expression of Huh7 cells in NT and sgA2B1 groups. (n = 3). Figure S5. Heatmap of the correlation between A2B1 expression and immune infiltration in HCC patients. Table S1. The detail of gene expression profiles of hepatocellular carcinoma. Table S2. Sequences for quantitative real-time PCR, PCR and sgRNA. Table S3. Comparison of differences in clinical information between patients grouped by high and low hnRNPA2B1 expression in the CHCC-HBV dataset. Table S4. Relative Quantification of metabolites in NT and sgA2B1 HuH7 cells using high-performance liquid chromatography/mass spectrometry (HPLC/MS) and gas chromatography/MS (GC/MS). [file 12967_2023_4704_MOESM1_ESM.docx]

**Additional File 1**


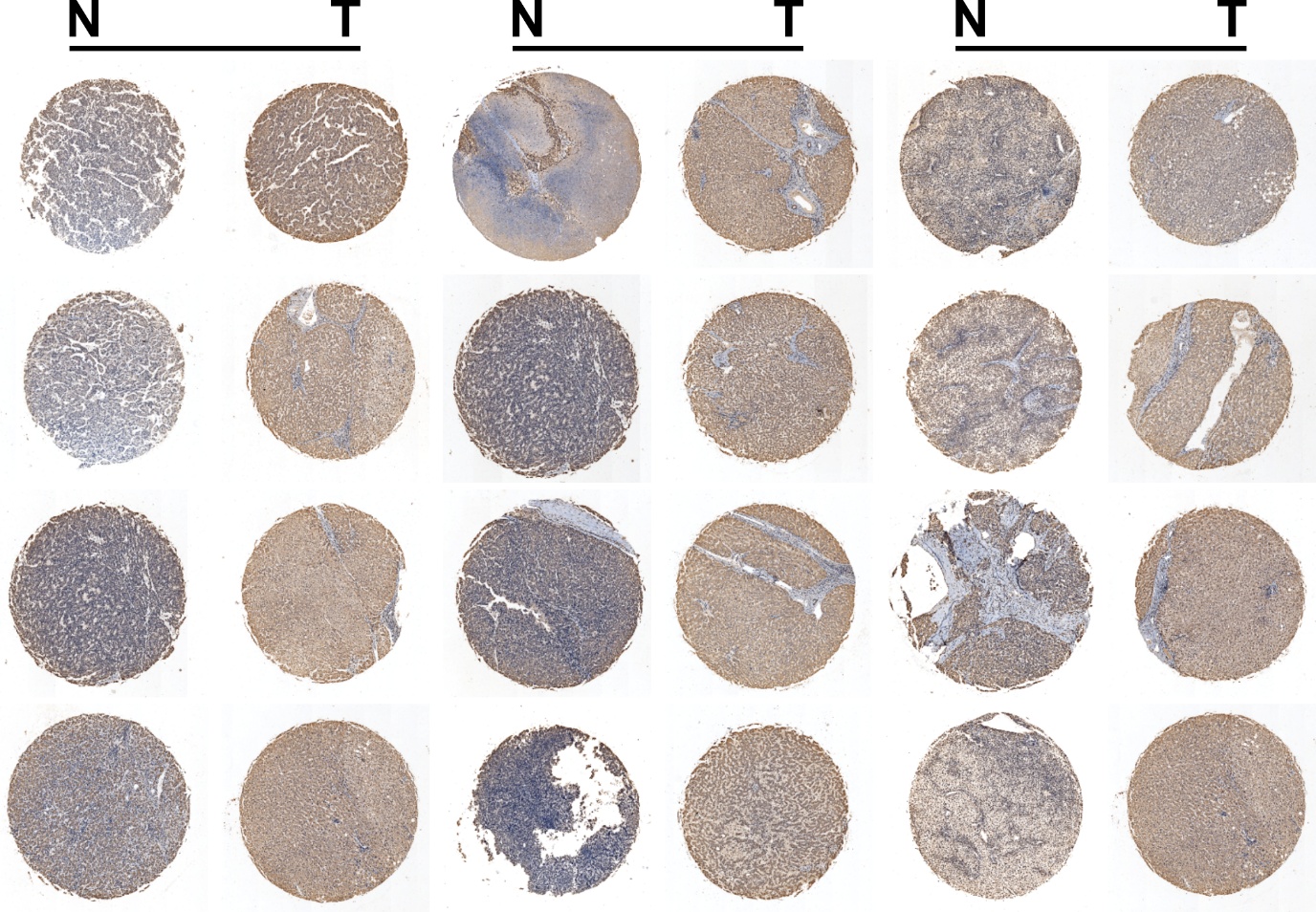


**Figure S1.** Representative immunohistochemistry images of hnRNPA2B1 expression in tissue microarrays of different HCC patients. T represents tumor tissues; N represents paired adjective non-tumor tissues.


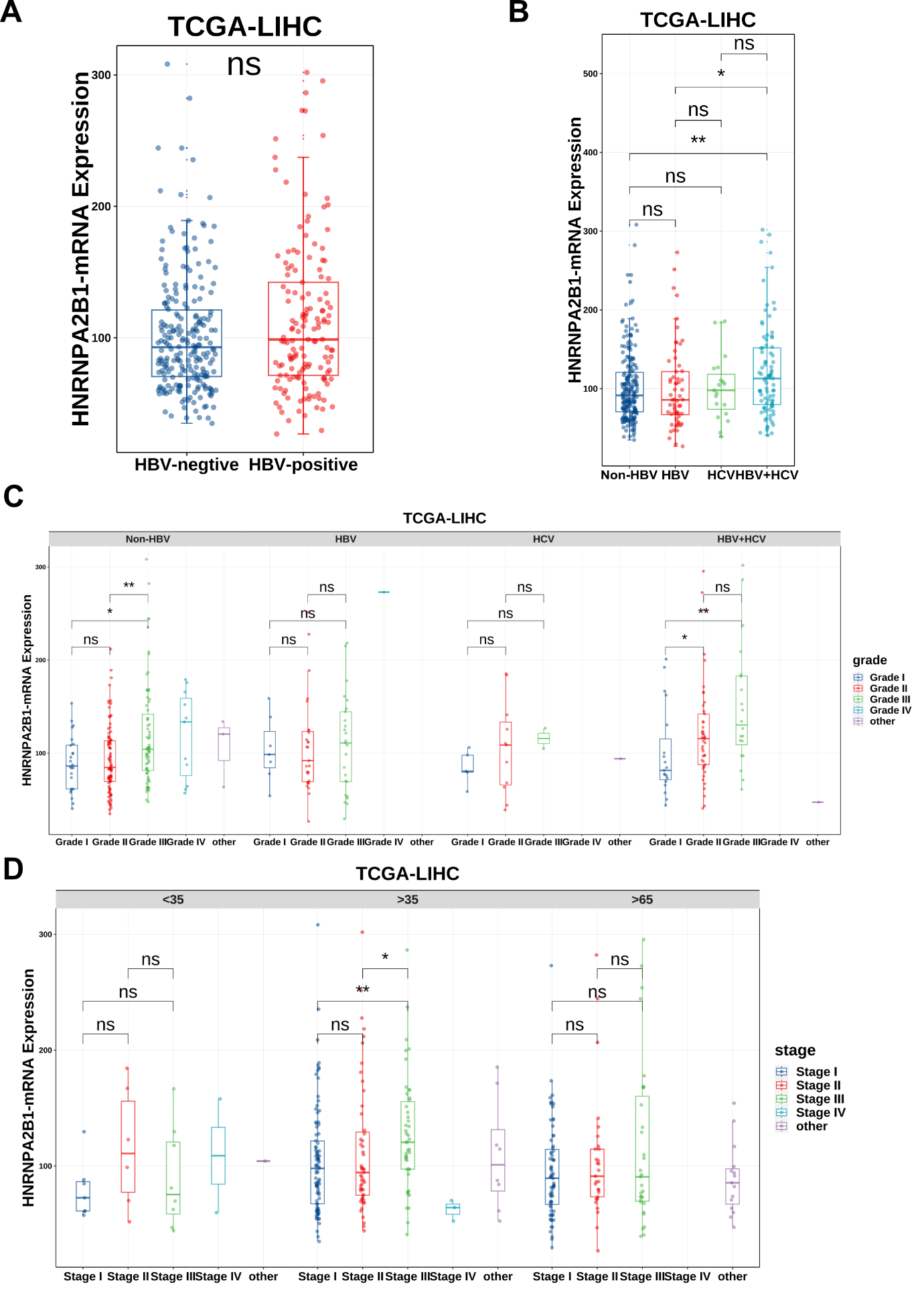


**Figure S2.** Correlation between of hnRNPA2B1 mRNA levels and HCC patients with HBV/HCV infection based on TCGA-LIHC database. (A) The relationship between hnRNPA2B1 mRNA levels and HCC patients

with or without HBV (Non-HBV) infection. (B) The relationship between hnRNPA2B1 mRNA levels and HCC patients with Non-HBV or HBV/HCV infection. (C, D) Correlation of hnRNPA2B1 RNA level, with patients at different clinical malignant grades, HBV/HCV infection, or age.

**Figure S3.** Heat map of differentially expressed gene expression of NT Huh7 cells and sgA2B1 Huh7 cells. (n=3).


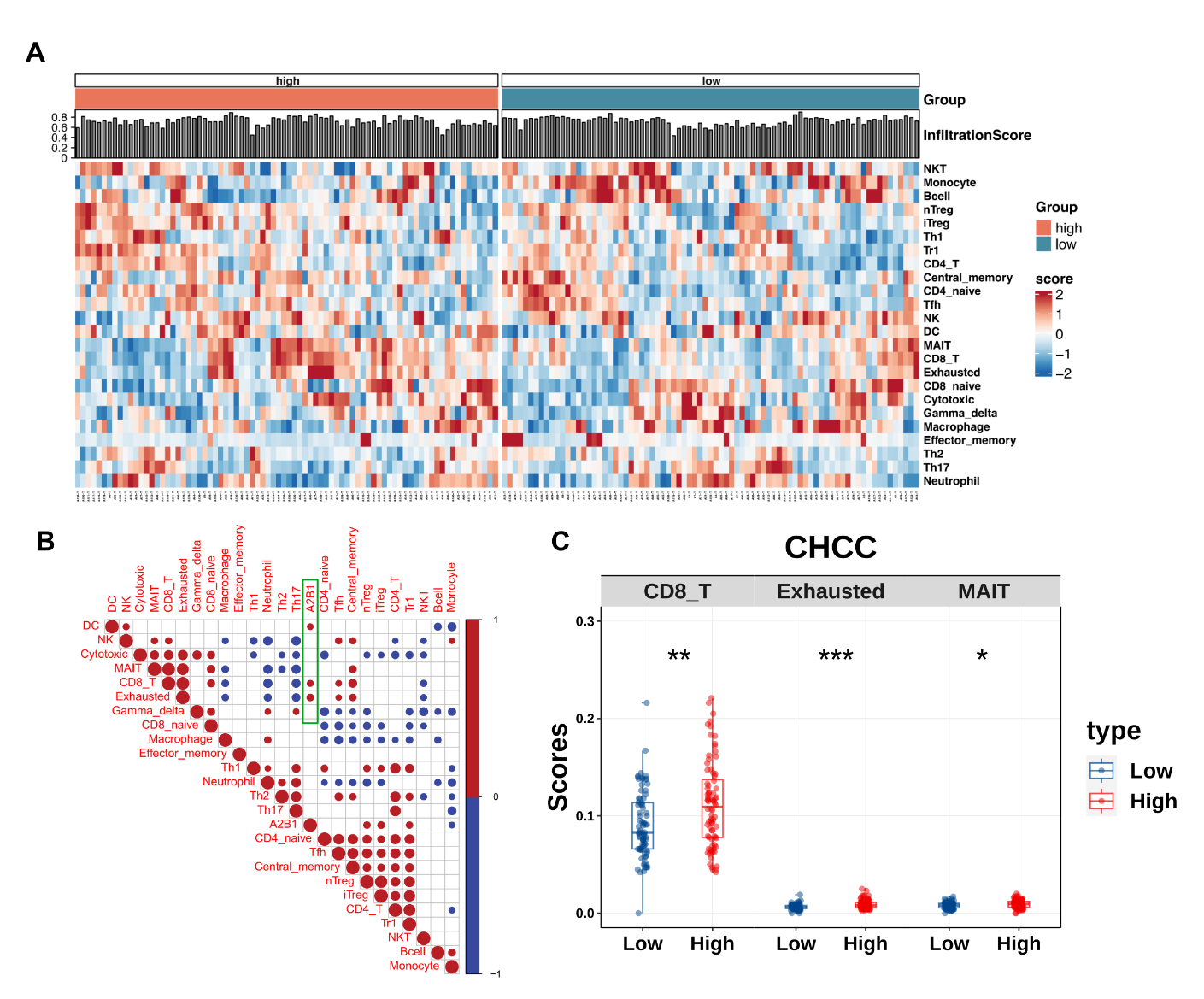


**Figure S4.** Correlation between A2B1 levels and immune infiltration in HCC cohort. A. Heatmap shows the Immune cell infiltration between high- and low-A2B1 mRNA level groups of the CHCC cohort. The median value of A2B1 expression was used to divide the high and low groups. B. Correlation analysis between hnRNPA2B1 expression and immune cell infiltration using Spearman's correlation tests. C. CD8 -T cells, Exhausted, and MAIT cell infiltration between high- and low-A2B1 mRNA level groups of the CHCC cohort.

**
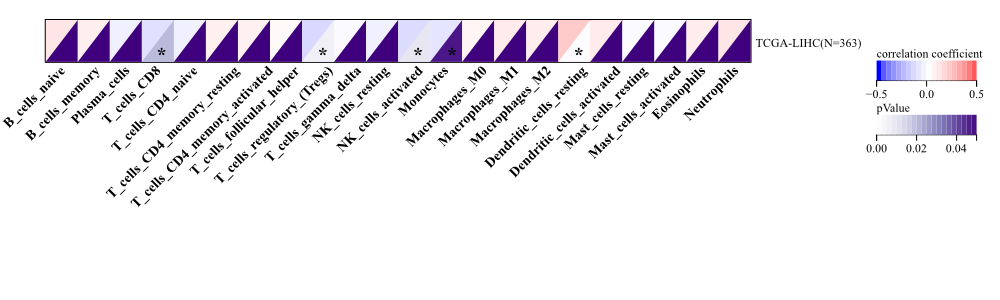
**

**Figure S5.** Heatmap of the correlation between A2B1 expression and immune infiltration in HCC patients.

**2. Supplementary Table:**

**Table S1. The detail of gene expression profiles of hepatocellular carcinoma.**

| Dataset | Website | Normal (n) | Tumor (n) |
| --- | --- | --- | --- |
| LIHC ( TCGA) | https://portal.gdc.cancer.gov/projects/TCGA-LIHC | 50 | 374 |
| CHCC-HBV | https://www.cell.com/cell/fulltext/S0092-8674(19)31003-7#supplementaryMaterial | 159 | 159 |
| GSE14520 | https://www.ncbi.nlm.nih.gov/geo/query/acc.cgi?acc=GSE14520 | 220 | 225 |
| GSE25097 | https://www.ncbi.nlm.nih.gov/geo/query/acc.cgi | 243 | 268 |
| GSE226544 | https://www.ncbi.nlm.nih.gov/geo/query/acc.cgi?acc=GSE226544 | 3 | 3 |

**Table S2.** Comparison of the differences in clinical information between patients grouped by high and low hnRNPA2B1 expression in the CHCC-H


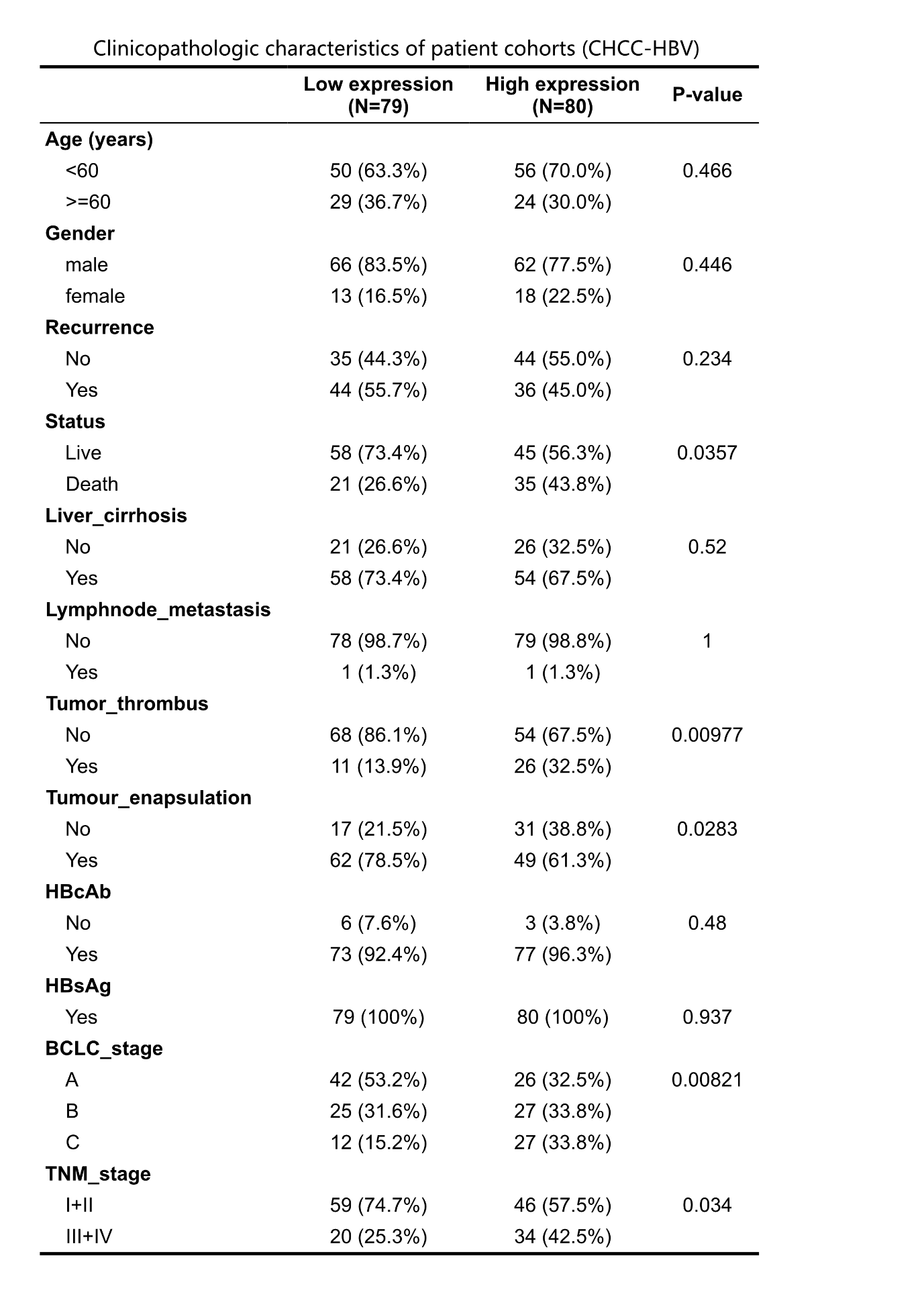


**Table S3.** Sequences for quantitative real-time PCR, PCR and sgRNA

| Name |  | Primer Sequence 5’-3’ | Description |
| --- | --- | --- | --- |
| β-actin | F | CACCATTGGCAATGAGCGGTTC | qRT-PCR |
|  | R | AGGTCTTTGCGGATGTCCACGT |  |
| PCK1 | F | AAAACGGCCTGAACCTCTCG |  |
|  | R | ACACAGCTCAGCGTTATTCTC |  |
| PCK2 | F | GCCATCATGCCGTAGCATC |  |
|  | R | AGCCTCAGTTCCATCACAGAT |  |
| FBP1 | F | CGCGCACCTCTATGGCATT |  |
|  | R | TTCTTCTGACACGAGAACACAC |  |
| G6PC | F | GTGTCCGTGATCGCAGACC |  |
|  | R | GACGAGGTTGAGCCAGTCTC |  |
| HK1 | F | GCTCTCCGATGAAACTCTCATAG |  |
|  | R | GGACCTTACGAATGTTGGCAA |  |
| HK2 | F | GAGCCACCACTCACCCTACT |  |
|  | R | CCAGGCATTCGGCAATGTG |  |
| PFKL | F | GTACCTGGCGCTGGTATCTG |  |
|  | R | CCTCTCACACATGAAGTTCTCC |  |
| PKM | F | ATGTCGAAGCCCCATAGTGAA |  |
|  | R | TGGGTGGTGAATCAATGTCCA |  |
| hnRNPA2B1 | sgRNA-F | CACCGACTCTCCCATCAATTGAATG | Homo sapiens |
|  | sgRNA-R | AAACCATTCAATTGATGGGAGAGTC |  |
|  | OE-F | ATTGATGGGAGAGTAGTTGAGCC |  |
|  | OE-R | AATTCCGCCAACAAACAGCTT |  |
| PCK1 | sgRNA-F | CACCGTGCTAAGGGGCACGAATGTG |  |
|  | sgRNA-R | AAACCACATTCGTGCCCCTTAGCAC |  |
|  | OE-F | TAGAAGATTCTAGAGCTAGCGAATTCGCCACCATGCCTCCTCAGCTGCAAAACGGCC |  |
|  | OE-R | ATCGCAGATCCTTGCGGCCGCGGATCCTTACATCTGGCTTATTCTTTGCTTCAAG |  |
| hnRNPA2B1 | sgRNA1-F | CGGAGGTCTTTCTCATCTCG | Mus musculus |
|  | sgRNA1-R | CGAGATGAGAAAGACCTCCG |  |
|  | sgRNA2-F | GTGAAGCGACTGAGTCCGCGA |  |
|  | sgRNA2-R | TCGCGGACTCAGTCGCTTCAC |  |
| PCK1 | sgRNA1-F | GCAATGGTGTGGAGAGAGGC |  |
|  | sgRNA1-R | GCCTCTCTCCACACCATTGC |  |
|  | sgRNA2-F | ACTTCCTCACTGCCTGGGGC |  |
|  | sgRNA2-R | GCCCCAGGCAGTGAGGAAGT |  |
| F:Former | R: Reverse |  |  |

**Table S4.** Relative levels of intracellular metabolites of gluconeogenesis in NT Huh7 and sgA2B1 Huh7.
